# Anti-IgE Treatment for Disorders Other Than Asthma

**DOI:** 10.3389/fmed.2017.00152

**Published:** 2017-09-21

**Authors:** Jeffrey Stokes

**Affiliations:** ^1^Division of Allergy, Immunology and Pulmonary Medicine, Department of Pediatrics, Washington University in St. Louis School of Medicine, St. Louis, MO, United States

**Keywords:** omalizumab, urticaria, asthma, immunotherapy, allergy

## Abstract

Immunoglobulin E (IgE) plays a key role in the pathogenesis of many allergic diseases. Thus, IgE-mediated immunologic pathways are an attractive target for intervention in allergic diseases. Omalizumab is a recombinant humanized monoclonal antibody that binds IgE and has been used treat allergic asthma for over a decade. Currently, omalizumab is approved for the treatment of both allergic asthma and chronic spontaneous urticaria. Since IgE plays a critical role in other allergic diseases, anti-IgE therapy has been evaluated in other allergic diseases in small clinical trials and case reports. Omalizumab has demonstrated efficacy in treating allergic rhinitis, atopic dermatitis, physical urticarias, mast cell disorders, food allergy, and other allergic diseases. In addition, the use of omalizumab with conventional allergen immunotherapy improves both safety and effectiveness.

## Immunoglobulin E (IgE) Background

Immunoglobulin E is responsible for the pathogenesis of many allergic diseases including asthma. The primary role of IgE is defense against parasitic disease such as helminthes and protozoa (1). IgE, like all immunoglobulins, is composed of two light chains and two identical heavy chains (2). The heavy chain for IgE is epsilon. IgE is a monomer and consists of four constant regions. The constant region, C-epsilon-3, binds to both the low- and high-affinity IgE receptor. Production of IgE requires two signals between T cells and B cells to override the default production of IgM by plasma cells (3). The first signal involves interleukin-4 (IL-4) and interleukin-13 (IL-13) release by T helper type 2 (Th2) cells, mast cells, and basophils interacting with their respective receptors on the B cell. The second signal is the interface between the B cell CD40 and the T cell CD40 ligand (CD154).

Immunoglobulin E functions *via* its high- and low-affinity receptors on mast cells, basophils, and other cells (4). The high-affinity receptor for IgE is Fc-epsilon-RI expressed on mast cells and basophils. When bound with IgE subsequent cross-linking leads to activation of the cell and release of preformed mediators and the production of other inflammatory cytokines. The inflammatory mediators released by mast cells and basophils include histamine, tryptases, tumor necrosis factor-alpha, leukotrienes, and prostaglandins. In addition, the production of the Th2 cytokines IL-4, IL-5, and IL-13 further initiate late-phase inflammation and promotes more IgE production. The low-affinity receptor Fc-epsilon-RII (CD23) in its inducible form is present on B cells, T cells, dendritic cells, monocytes, macrophages, neutrophils, eosinophils, intestinal epithelial cells, and platelets (5, 6). The low-affinity receptor helps regulates IgE synthesis and has a role in antigen presentation (5, 6).

## Anti-IgE Antibody (Omalizumab)

Omalizumab is a recombinant humanized immunoglobulin (IgG1) monoclonal antibody that binds IgE with high affinity developed for the treatment of allergic diseases (7). Omalizumab binds to the same C-epsilon-3 region that interacts with the IgE receptors forming complexes with free IgE preventing its interaction with these receptors (8). The omalizumab-IgE complexes are subsequently cleared by the hepatic reticuloendothelial system. Omalizumab is specific to IgE and does not bind to IgG or IgA. An important property of omalizumab is that it cannot bind to the IgE receptors or to IgE already attached to Fc-epsilon-RI, and therefore does not interact with cell-bound IgE or activate mast cells or basophils. Administration of omalizumab results in a rapid and significant decrease in free IgE levels. Due to this dramatic decrease in circulating IgE omalizuamb subsequently decreased the expression of the high affinity FcεRI receptor on the surface of both mast cells and basophils (9).

## Omalizumab’s Effect on Eosinophils

In a pooled analysis of over 2,200 patients, omalizumab treatment reduced blood eosinophil counts which correlated with the reduction seen in free IgE (10). In asthmatic patients, two studies evaluated the effect of omalizumab on sputum eosinophils and bronchial biopsies. The first study by Djukanović et al. examined induced sputum and bronchial biopsies on 45 moderate to severe asthma patients with baseline sputum eosinophils ≥2% (11). Omalizumab treatment for 16 weeks reduced mean sputum eosinophils from 6.6 to 1.7%, while the reduction in the placebo group was only 8.5 to 7.0%. In the submucosal bronchial biopsies median eosinophil counts decreased from 8.0 to 1.5 cells/mm^2^ with omalizumab treatment while the counts were 6.3 to 6.4 cells/mm^2^ in the placebo group. There was a weak correlation with the reduction in submucosal eosinophils and reduction in cells producing IL-4. Van Rensen et al. studied the effects of omalizumab on allergen challenge with 25 atopic asthmatics (12). In their study, omalizumab decreased sputum eosinophils from 4 to 0.5% and bronchial biopsy eosinophil count from 15 to 2 cells/0.1 mm^2^.

One proposed mechanism for the reduction of eosinophils is by inducing eosinophil apoptosis. Nineteen patients with allergic asthma were treated 3 months of omalizumab (13). A marker of eosinophil apoptosis (annexin V) was increased in those patients treated with omalizamab and annexin-positive eosinophils were increased compared to baseline. In addition, cellular production of GM-CSF, used for eosinophil growth and survival, was decreased.

## Asthma

In the United States and worldwide, omalizumab is approved for use in patients 6 years of age and older with moderate-to-severe persistent perennial asthma (14). In patients with moderate-to-severe asthma, multiple studies have demonstrated that treatment with omalizumab (compared with placebo) decreases the incidence of exacerbations and significantly reduces the dose of inhaled or oral glucocorticoids required to control symptoms. In two studies of pooled data omalizumab reduced emergency room, asthma-related outpatient visits, and hospitalizations (15, 16). Several studies have examined omalizumab therapy in children. These studies demonstrated reduced exacerbations, asthma symptoms, inhaled corticosteroid (ICS) doses, daily systemic corticosteroid dose, and hospitalizations with omalizumab therapy (17–19). In 2014, a meta-analysis of 25 randomized trials of patients with moderate or severe asthma requiring inhaled glucocorticoids, omalizumab reduced the risk of experiencing an exacerbation from 26 to 16% over 16 to 60 weeks of treatment (20). In addition, omalizumab reduced the risk of hospitalization for asthma from 3 to 0.5% over 28 to 60 weeks as well as decreased the amount of ICSs needed for asthma control. In this analysis, subjects receiving omalizumab were more likely to be able to completely withdraw inhaled glucocorticoids compared with those receiving placebo (40 versus 21%). However, omalizumab did not appear to increase the likelihood that subjects could discontinue oral glucocorticoids or consistently improve lung function in this meta-analysis.

## Predictors of Response to Omalizumab in Asthma Patients

Asthma is a heterogenous disease with several different phenotypes. When based on inflammatory markers the Th_2_ pattern is the most common. The EXTRA omalizumab study evaluated 850 patients with severe perennial allergic asthma divided into low- and high-biomarker groups (F_ENO_, blood eosinophils, and serum periostin) (21). Patients in the high eosinophil group (≥260/μL) had a greater reduction in exacerbations with omalizumab treatment than those in the low group (<260/μL). In a pediatric trial of omalizumab to prevent fall asthma exacerbations during the run in period patients those who had an exacerbation had a higher serum eosinophil count (350 cells/μL) compared to those without an exacerbation (280 cells/μL) (22). A 24-week, multicenter, parallel group, double-blind, randomized, placebo controlled trial on patients with symptomatic asthma despite ICSs evaluated exacerbation rates with 6 months of anti-IgE therapy (23). In a subgroup analysis, patients with a baseline eosinophil count of ≥300/mL treated with omalizumab had a reduction in protocol-defined exacerbations by nearly 60% compared to placebo. When using the exacerbations defined by ATS/ERS criteria, the rate was reduced by 45% with omalizumab treatment. In those patients with the low baseline eosinophil counts, no improvement was noted in protocol defined exacerbation. There are limited characteristics that predict a positive response to omalizumab therapy for asthma but findings suggest that patients with a higher baseline serum eosinophil count may have a better clinical outcome.

## Chronic Urticaria

Urticaria and/or angioedema that occurs daily or near daily for more than 6 weeks has been termed chronic idiopathic urticaria (CU) or chronic spontaneous urticaria (CSU). First-line therapy, oral H1 antihistamines are effective for 50–60% of these patients (24). The initial proof-of-concept study involved 90 patients with antihistamine refractory CSU treated with a single administration of three different doses of omalizumab, 75, 300, or 600 mg versus placebo (25). Only the 300 and 600 mg doses demonstrated improvement in the urticaria scores 4 weeks after treatment and there was not a significant difference in efficacy between these doses. This led to three large, phase III, randomized, double-blind, placebo controlled studies, Asteria I, Asteria II, and Glacial (26–28). These studies evaluated patients aged 12–75 with CSU that was refractory to oral H1-antihistamines.

In the Asteria I trial, 318 patients were randomized to one of three different doses of omalizumab (75, 150, and 300 mg) or placebo every 4 weeks for 24 weeks after failing licensed doses of H1-antihistamine therapy (26). Within the first week, the 300 mg dose improved urticaria compared to placebo. At 12 weeks, all three omalizumb doses significantly reduced patient’s symptoms at 12 weeks compared to placebo. By week 12, 52% of the high-dose omalizumab patients were well controlled and 36% completely controlled. In addition, the 300 mg dose improved associated angioedema symptoms.

The Asteria II trial was similar in design with the same doses of omalizumab or placebo and enrolled 323 patients with CSU who remained symptomatic despite H1-antihistamine therapy (27). In this study, patients were treated for only 12 weeks compared to 24 in the Asteria I. At the end of the study, the patients on 150 and 300 mg doses of omalizumab demonstrated significant improvements in symptom scores and number of hives compared to placebo with 53% of the group receiving 300 mg of omalizumab becoming hive free and 44% free from both hives and itching.

The Glacial trial had a significant difference compared to the two Asteria trials. These patients all failed H1-antihistamines up to four times licensed doses plus patients were allowed to have been on an H2-blocker and/or leukotriene antagonist (28). In this study, 335 patients were randomized to either 300 mg of omalizumab or placebo monthlym for 24 weeks of treatment with 16-week follow-up. This study demonstrated the effectiveness of 300 mg omalizumab monthly in reducing urticarial lesions and symptoms after 12 weeks of therapy which was sustained for the 24 weeks of therapy.

In a review of over 900 patients with CSU symptomatic despite conventional therapy omalizumab improved symptoms in 65% with complete resolution in 40%. The improvement was noted in a just a few days in a subset of these patients (29). Omalizumab has also improved symptoms in patients with different types of physical urticarias, including solar, cold, localized heat, cholinergic, dermatographic, and pressure (30–37). In successfully treated patients with CSU or with physical uriticaria discontinuing therapy may lead to, relapse within a few weeks. Retreatment with omalizumab was effective resolving those symptoms (38).

Several associations such as the American Academy of Allergy, Asthma & Immunology, American College of Allergy, Asthma & Immunology, European Academy of Allergy and Clinical Immunology, Global Allergy and Asthma European Network, European Dermatology Forum, and the World Allergy Organization have recommended the use of omalizumab for CU in their urticaria guidelines (39, 40). In March of 2014, the US Food and Drug Administration approved the use of omalizumab in chronic urticaria patients 12 years and older who remain symptomatic despite H1 antihistamines.

The exact mechanisms of how omalizumab works in CU are unclear. In a subset of patients, IgG autoantibodies against FcεRI, IgE, or both may exist (41). Since omalizumab decreases the free IgE available with subsequent down-regulation of the FcεRI receptor, it was natural to postulate that omalizumab’s effects might be due to decreasing the targets for these autoantibodies. However, no differences in effectiveness have been found in patients with or without the autoantibodies. Furthermore, analysis of previous data suggests that it takes time to significantly decrease the expression of the high affinity IgE receptors on either basophils (2 weeks) or mast cells (10 weeks); whereas a therapeutic effect within 1 week is noted in some patients (42, 43). Another possible mechanism is that IgE antibodies against autoallergens are present, and omalizumab reduces the level of these autoantibodies. Similar autoantibodies have been noted in patients with systemic lupus erythematosus contributing to the pathogenesis of that disease and similar autoimmune disorders (44).

## Atopic Dermatitis

Atopic dermatitis is another potential allergic target for anti-IgE therapy. Atopic dermatitis has been treated with omalizumab in several case series of both adult and pediatric patients, but results have been mixed. In a small series of seven pediatric patients with severe atopic dermatitis clinical improvement was noted 3 to 6 months after starting therapy and all patients had improvement after 12 months of therapy (45). Heil et al. performed a randomized, placebo-controlled, double-blind pilot study on 20 atopic dermatitis patients (46). Patients were randomized to omalizumab (13) or placebo (7) treatments for 16 weeks. At the end of the study, omalizumab reduced free IgE, surface IgE, and FceRI expression. Despite these changes, no significant improvement was noted with omalizumab therapy on clinical symptoms of atopic dermatitis. Iyengar et al. performed another randomized, double-blind, placebo-controlled study of eight patients (four omalizumab, four placebo) with severe refractory atopic dermatitis (47). After 24 weeks of therapy, omalizumab decreased levels of allergic inflammatory mediators but clinical symptoms were comparable between the omalizumab and placebo groups. A recent meta-analysis included the previously mentioned randomized studies as well as 13 case series evaluating the effectiveness of omalizumab in treating atopic dermatitis (48). There was no concrete evidence that omalizumab was effective in treating atopic dermatitis. Despite that conclusion, it was noted that 43% of patients improved clinically with omalizumab. There may be specific types of patients who are more responsive than others based on multiple variables, age, baseline IgE level, atopic status, or presence of filaggrin mutation (49).

## Allergic Rhinitis

Allergic rhinitis is another potential target for anti-IgE therapy. An early study in the United States evaluated 536 ragweed allergic patients at 25 different sites (50). This randomized, double-blind, placebo-controlled study evaluated several doses of omalizumab (50, 150, and 300 mg) and placebo administered every 3–4 weeks just prior to and during ragweed season. Patients treated with 300 mg of Omalizumab had significantly lower rhinitis symptoms. Those treated with the 300 mg dose of omalizumab had better quality of life scores than the other groups and did not decline during the peak ragweed season. A follow-up open-labeled study of 300 mg of omalizumab therapy every 3–4 weeks demonstrated that the therapy is well tolerated without any significant immunologic reactions (51). Further studies demonstrated omalizumab’s effectiveness in reducing symptoms and rescue medication usage in patients with allergic rhinitis to ragweed, birch, cedar, and perennial allergens (52–58).

A meta-analysis published in 2014 retrieved 352 citations with 78 articles eligible for review (59). Of those studies, 11 qualified for evaluation with a total of 2,870 patients treated for seasonal or perennial allergic rhinitis. Omalizumab significantly reduced both daily nasal symptoms and daily nasal rescue medication usage. No significant adverse events were reported.

## Allergen Immunotherapy

Allergen immunotherapy has been used for 100 years for the management of allergic disorders and is the only antigen-specific immunomodulatory treatment. The addition of omalizumab to standard maintenance-dose immunotherapy was evaluated in 221 pediatric patients sensitized to birch and grass pollen (60). During birch season, the addition of omalizumab reduced symptoms by 48% compared to birch SCIT alone. Similar results were seen in grass season, with a 57% decrease in symptoms with the addition of omalizumab to grass immunotherapy compared to grass immunotherapy alone. When these findings were further analyzed for the grass pollen–allergic children, the groups treated with omalizumab plus immunotherapy had significantly diminished rescue medication use and number of symptomatic days compared to omalizumab or immunotherapy alone (61). Casale et al. evaluated omalizumab starting 9 weeks before rush immunotherapy followed by 12 weeks of therapy (62). Overall, in ragweed-allergic patients the combination of omalizumab and immunotherapy showed a significant improvement in severity scores during the ragweed season compared with those receiving immunotherapy alone after rush immunotherapy buildup. Overall, these findings demonstrate that combined treatment with omalizumab and immunotherapy is more effective than omalizumab or immunotherapy alone.

Immunotherapy for allergens carries a risk of anaphylaxis with each administration. In those patients treated with rush ragweed immunotherapy, omalizumab added to immunotherapy had fewer adverse events than those receiving immunotherapy alone. The addition of omalizumab to SCIT resulted in a decrease in risk of anaphylaxis caused by immunotherapy by fivefold (62). In asthma patients, the use of omalizumab in conjunction with SCIT resulted in fewer systemic reactions (13.5 versus 26.2% for placebo) (63). The use of omalizumab in conjunction with venom immunotherapy in a few patients demonstrated conflicting results in preventing systemic reactions caused by immunotherapy (64, 65). Recently, the use of oral immunotherapy for food allergy noted benefit in patients with milk, egg, and peanut allergy. The data suggest that omalizumab may facilitate oral desensitization to peanut and milk (66, 67). The addition of omalizumab has allowed some children to successfully receive oral immunotherapy to multiple foods simultaneously, including milk, egg, peanut, wheat, soy, and tree nuts (66–69). The addition of omalizumab to oral immunotherapy for milk not only improved safety but also decreased basophil activation (70).

## Food Allergy

Food allergies affect about 6% of children younger than 3 years of age and 2% of adults, with 1.5 million suffering from peanut allergy in the United States. An early double-blind, placebo-controlled, randomized trial in 84 peanut allergic patients evaluated three doses of another humanized mAb against IgE, TNX-901 (71). All groups, including the placebo group, had a greater threshold of peanut tolerability, but only the high-dose TNX-901 group significantly improved from tolerating about 1/2 peanut to more than 8 peanuts at the end of therapy. Despite this 25% of the high-dose group had no improvement with therapy. A more recent attempt at a phase II, multicenter, randomized, double-blind, placebo-controlled, parallel-group trial was designed to assess the efficacy of omalizumab in peanut allergic patients (72). Due to concern of severe anaphylactic reactions during the qualifying food challenges before therapy, only 14 patients (9 omalizumab and 4 placebo) completed treatment. Of these, four (44.4%) omalizumab-treated subjects compared to one (20%) placebo-treated subjects could tolerate >1,000 mg peanut flour after 24 weeks, but this difference was not significant. Both of these studies suggest that omalizumab may be beneficial for food allergy, but the findings are not conclusive.

## Eosinophilic Esophagitis

Eosinophilic esophagitis is another potential target for anti-IgE therapy. In two case studies of patients with eosinophilic esophagitis and multiple food allergies, the addition of omalizumab to the patient’s standard therapy reduced symptoms of eosinophilic esophagitis but did not improve endoscopic and histologic changes (73). A prospective randomized, double-blind, placebo-controlled trial of omalizumab therapy monthly for 16 weeks in 30 eosinophilic esophagitis patients who were either refractory to or relapsed after topical corticosteroids found no improvements in either esophageal eosinophil counts or symptom scores (74). In an open label study, omalizumab was administered for 12 weeks to 15 subjects with long standing EoE, only 5 of the subjects had histological and clinical improvement after 3 months of treatment (75). Omalizumab induced remission of EoE was limited to those subjects with low baseline peripheral blood absolute eosinophil counts (<450 cells/μl). Foroughi et al. examined the effect of omalizumab on assorted eosinophilic gastrointestinal disorders (76). Of the nine subjects, eight had multiple areas of eosinophilic disease with seven having at least the esophagus involved. Patients were treated with omalizumab every 2 weeks for 16 weeks. There was a non-significant decrease in eosinophils present in the duodenum and stomach while the esophageal eosinophils were unchanged. Despite this lack of histological changes, symptom scores were significantly decreased by 70% at the end of the study. No effect on T-cell function was noted in those patients treated with omalizumab (77). Overall, these studies suggest that for select patients with eosinophilic-based GI diseases anti-IgE therapy may be effective, especially those with low blood eosinophil counts.

## Allergic Bronchopulmonary Aspergillosis (ABPA)

Allergic bronchopulmonary aspergillosis is associated with pediatric cystic fibrosis and adult asthma. One review on children with cystic fibrosis and ABPA analyzed 8 cases in 13 children (78). Treatment with omalizumab improved lung functions and reduced respiratory symptoms and systemic corticosteroid use. A Cochrane meta-analysis attempted to evaluate the effects of omalizumab for ABPA in patients with cystic fibrosis, but concluded that there were no studies that met inclusion criteria (79). A retrospective analysis of four adult stage IV ABPA patients (corticosteroid dependent) treated with omalizumab 375 mg every 2 weeks for 12 months found it was steroid sparing and reduced inflammatory markers and symptom scores, even with elevated IgE levels (80).

## Nasal Polyps

Nasal polyps are frequently associated with eosinophilic inflammation and local production of IgE. In a randomized, double-blind, placebo-controlled study, patients with nasal polyps and comorbid asthma were treated with either omalizumab (16) or placebo (8) for 16 weeks (81). In the omalizumab-treated group, nasal polyp size decreased by both endoscopy and CT scan assessment, regardless of allergic status. Only the omalizumab-treated patients had a significant improvement in their nasal and asthma symptom scores. A similar study of patients with nasal polyps who were continued on their medical regimen (nasal corticosteroids, leukotriene modifiers, and as needed courses of prednisone and antibiotics) found that the addition of omalizumab decreased nasal polyp size but had no significant effect on symptoms compared to the placebo group (82). A retrospective analysis of eight subjects demonstrated that omalizumab after polypectomey may reduce the severity of nasal polyp recurrence (83).

## Other Diseases

A recent review on the use of omalizumab in mast cell disorders noted case studies of patients with mastocytosis, cutaneous mastocytosis, venom anaphylaxis, and mast cell activation syndrome showed improvement with omalizumab therapy (84). Case reports and small studies have noted the benefit of omalizumab treatment in Churg–Strauss Syndrome, bullous pemphigoid, Kimura’s disease, aspirin-exacerbated respiratory disease, recurrent anaphylaxis, laryngeal angioedema, chronic eosinophilic pneumonia, drug allergy, and vernal keratoconjunctivitis (85–93).

## Conclusion

Omalizumab is the first immune modifier to be approved for the treatment of allergic diseases (Table 1). The excellent strength of evidence for the effectiveness of omalizumab in allergic asthma and chronic urticaria have resulted in the FDA approval for use in those diseases. While evidence points to omalizumab’s effectiveness in allergic rhinitis, and as an adjunct to allergen immunotherapy, due to costs or dosing limitations omalizumab will unlikely be widely used for those instances. Large clinical trials are needed for omalizumab and other anti-IgE strategies to treat the other allergic diseases were the evidence is not as strong.

**Table 1:** Clinical benefit with omalizumab therapy.

Strong evidence	Allergic asthma Chronic urticaria
Good evidence	Allergic rhinitis Allergen immunotherapy (inhalants)
Fair evidence	Atopic dermatitis Food allergy Oral immunotherapy (foods) Mast cell disorders
Weak evidence	Eosinophilic gastrointestinal diseases Allergic bronchopulmonary aspergillosis Nasal polyps

## Author Contributions

The author confirms being the sole contributor of this work and approved it for publication.

## Conflict of Interest Statement

The author declares that the research was conducted in the absence of any commercial or financial relationships that could be construed as a potential conflict of interest.

## References

[1] Lynch NR Hagel IA Palenque ME Di Prisco MC Escudero JE Corao LA Relationship between helminthic infection and IgE response in atopic and nonatopic children in a tropical environment. J Allergy Clin Immunol (1998) 101:217. 10.1016/S0091-6749(98)70386-0 9500755

[2] Schroeder HW Jr Cavacini L. Structure and function of immunoglobulins. J Allergy Clin Immunol (2010) 125:S41–52. 10.1016/j.jaci.2009.09.046 20176268 PMC3670108

[3] Vercelli D Immunobiology of IgE. 7th ed In: Adkinson N Bochner BS Busse WW, editors. Middleton’s Allergy: Principles and Practice. Mosby Elsevier (2009).

[4] Stone KD Prussin C Metcalfe DD. IgE, mast cells, basophils, and eosinophils. J Allergy Clin Immunol (2010) 125:S73–80. 10.1016/j.jaci.2009.11.017 20176269 PMC2847274

[5] Geha RS Jabara HH Brodeur SR. The regulation of immunoglobulin E class-switch recombination. Nat Rev Immunol (2003) 3:721. 10.1038/nri1181 12949496

[6] Tsicopoulos A Joseph M The role of CD23 in allergic disease. Clin Exp Allergy (2000) 30(5):602 10.1046/j.1365-2222.2000.00871.x 10792350

[7] Fick RB Jr. Anti-IgE as novel therapy for the treatment of asthma. Curr Opin Pulm Med (1999) 5:76. 10.1097/00063198-199901000-00013 10813254

[8] Easthope S Jarvis B. Omalizumab. Drugs (2001) 61:253. 10.2165/00003495-200161020-00008 11270941

[9] MacGlashan DW Bochner BS Adelman DC Jardieu PM Togias A McKenzie-White J Down-regulation of FcεRI expression on human basophils during in vivo treatment of atopic patients with anti-IgE antibody. J Immunol (1997) 158:1438–45. 9013989

[10] Massanari M Holgate ST Busse WW Jimenez P Kianifard F Zeldin R. Effect of omalizumab on peripheral blood eosinophilia in allergic asthma. Respir Med (2010) 104(2):188–96. 10.1016/j.rmed.2009.09.011 19846286

[11] Djukanović R Wilson SJ Kraft M Jarjour NN Steel M Chung KF Effects of treatment with anti-immunoglobulin E antibody omalizumab on airway inflammation in allergic asthma. Am J Respir Crit Care Med (2004) 170:583–93. 10.1164/rccm.200312-1651OC 15172898

[12] van Rensen EL Evertse CE van Schadewijk WA van Wijngaarden S Ayre G Mauad T Eosinophils in bronchial mucosa of asthmatics after allergen challenge: effect of anti-IgE treatment. Allergy (2009) 64(1):72–80. 10.1111/j.1398-9995.2008.01881.x 19076931

[13] Noga O Hanf G Brachmann I Klucken AC Kleine-Tebbe J Rosseau S Effect of omalizumab treatment on peripheral eosinophil and T-lymphocyte function in patients with allergic asthma. J Allergy Clin Immunol (2006) 117(6):1493–9. 10.1016/j.jaci.2006.02.028 16751018

[14] Package insert, Xolair (revision). (2017). Available from: https://www.gene.com/download/pdf/xolair_prescribing.pdf

[15] Corren J Casale T Deniz Y Ashby M Omalizumab, a recombinant humanized anti-IgE antibody, reduces asthma-related emergency room visits and hospitalizations in patients with allergic predicting response to omalizumab, and anti-IgE antibody, in patients with allergic asthma. J Allergy Clin Immunol (2003) 111:87–90. 10.1067/mai.2003.49 12532101

[16] Bousquet J Cabrera P Berkman N Buhl R Holgate S Wenzel S The effect of treatment with omalizumab, an anti-IgE antibody, on asthma exacerbations and emergency medical visits in patients with severe persistent asthma. Allergy (2005) 60:302–8. 10.1111/j.1398-9995.2004.00770.x 15679714

[17] Milgrom H Berger W Nayak A Gupta N Pollard S McAlary M Treatment of childhood asthma with anti-immunoglobulin E antibody (omalizumab). Pediatrics (2001) 108:E36. 10.1542/peds.108.2.e36 11483846

[18] Lanier B Bridges T Kulus M Taylor AF Berhane I Vidaurre CF. Omalizumab for the treatment of exacerbations in children with inadequately controlled allergic (IgE-mediated) asthma. J Allergy Clin Immunol (2009) 124:1210–6. 10.1016/j.jaci.2009.09.021 19910033

[19] Busse WW Morgan WJ Gergen PJ Mitchell HE Gern JE Liu AH Randomized trial of omalizumab (anti-IgE) for asthma in inner-city children. N Engl J Med (2011) 364:1005–15. 10.1056/NEJMoa1009705 21410369 PMC3093964

[20] Normansell R Walker S Milan SJ Walters EH Nair P Omalizumab for asthma in adults and children. Cochrane Database Syst Rev (2014) 1:CD003559 10.1002/14651858.CD003559.pub4 PMC10981784 24414989

[21] Hanania NA Wenzel S Rosén K Hsieh HJ Mosesova S Choy DF Exploring the effects of omalizumab in allergic asthma: an analysis of biomarkers in the EXTRA study. Am J Respir Crit Care Med (2013) 187(8):804–11. 10.1164/rccm.201208-1414OC 23471469

[22] Teach SJ Gill MA Togias A Sorkness CA Arbes SJ Jr Calatroni A Preseasonal treatment with either omalizumab or an inhaled corticosteroid boost to prevent fall asthma exacerbations. J Allergy Clin Immunol (2015) 136(6):1476–85. 10.1016/j.jaci.2015.09.008 26518090 PMC4679705

[23] Busse W Spector S Rosén K Wang Y Alpan O High eosinophil count: a potential biomarker for assessing successful omalizumab treatment effects. J Allergy Clin Immunol (2013) 132(2):485–6.e11. 10.1016/j.jaci.2013.02.032 23591271

[24] Khan DA. Alternative agents in refractory chronic urticaria: evidence and considerations on their selection and use. J Allergy Clin Immunol Pract (2013) 1:433–40.e1. 10.1016/j.jaip.2013.06.003 24565613

[25] Saini S Rosen KE Hsieh HJ Wong DA Conner E Kaplan A A randomized, placebo-controlled, dose-ranging study of single-dose omalizumab in patients with H1-antihistamine-refractory chronic idiopathic urticaria. J Allergy Clin Immunol (2011) 128:567–73. 10.1016/j.jaci.2011.06.010 21762974

[26] Saini SS Bindslev-Jensen C Maurer M Grob JJ Bülbül Baskan E Bradley MS Efficacy and safety of omalizumab in patients with chronic idiopathic/spontaneous urticaria who remain symptomatic on H(1) antihistamines: a randomized, placebo-controlled study. J Invest Dermatol (2015) 135(1):67–75. 10.1038/jid.2014.306 25046337 PMC4269803

[27] Maurer M Rosén K Hsieh HJ Saini S Grattan C Gimenéz-Arnau A Omalizumab for the treatment of chronic idiopathic or spontaneous urticaria. N Engl J Med (2013) 368:924–35. 10.1056/NEJMoa1215372 23432142

[28] Kaplan A Ledford D Ashby M Canvin J Zazzali JL Conner E Omalizumab in patients with symptomatic chronic idiopathic/spontaneous urticaria despite standard combination therapy. J Allergy Clin Immunol (2013) 132:101–9. 10.1016/j.jaci.2013.05.013 23810097

[29] Kaplan AP. Therapy of chronic urticaria: a simple, modern approach. Ann Allergy Asthma Immunol (2014) 112:419–25. 10.1016/j.anai.2014.02.014 24656924

[30] Metz M Bergmann P Zuberbier T Maurer M Successful treatment of cholinergic urticaria with anti-immunoglobulin E therapy. Allergy (2008) 63:247–9. 10.1111/j.1398-9995.2007.01591.x 18186820

[31] Sands MF Blume JW Schwartz SA Successful treatment of 3 patients with recurrent idiopathic angioedema with omalizumab. J Allergy Clin Immunol (2007) 120:979–81. 10.1016/j.jaci.2007.07.041 17931567

[32] Spector SL Tan RA. Effect of omalizumab on patients with chronic urticaria. Ann Allergy Asthma Immunol (2007) 99:190–3. 10.1016/S1081-1206(10)60644-8 17718108

[33] Kaplan AP Joseph K Maykut RJ Geba GP Zeldin RK. Treatment of chronic autoimmune urticaria with omalizumab. J Allergy Clin Immunol (2008) 122:569–73. 10.1016/j.jaci.2008.07.006 18774392

[34] Maurer M Altrichter S Bieber T Biedermann T Bräutigam M Seyfried S Efficacy and safety of omalizumab in patients with chronic urticaria who exhibit IgE against thyroperoxidase. J Allergy Clin Immunol (2011) 128:202–9.e5. 10.1016/j.jaci.2011.04.038 21636116

[35] Ferrer M Gamboa P Sanz ML Goikoetxea MJ Cabrera-Freitag P Javaloyes G Omalizumab is effective in nonautoimmune urticaria. J Allergy Clin Immunol (2011) 127:1300–2. 10.1016/j.jaci.2010.12.1085 21315432

[36] Metz M Altrichter S Ardelean E Kessler B Krause K Magerl M Anti-immunoglobulin E treatment of patients with recalcitrant physical urticaria. Int Arch Allergy Immunol (2011) 154:177–80. 10.1159/000320233 20733327

[37] Groffik A Mitzel-Kaoukhov H Magerl M Maurer M Staubach P Omalizumab – an effective and safe treatment of therapy-resistant chronic spontaneous urticaria. Allergy (2011) 66:303–5. 10.1111/j.1398-9995.2010.02472.x 21208221

[38] Metz M Ohanyan T Church MK Maurer M. Retreatment with omalizumab results in rapid remission in chronic spontaneous and inducible urticaria. JAMA Dermatol (2014) 150:288–90. 10.1001/jamadermatol.2013.8705 24477320

[39] Zuberbier T Aberer W Asero R Bindslev-Jensen C Brzoza Z Canonica GW The EAACI/GA(2) LEN/EDF/WAO guideline for the definition, classification, diagnosis, and management of urticaria: the 2013 revision and update. Allergy (2014) 69:868–87. 10.1111/all.12313 24785199

[40] Bernstein JA Lang DM Khan DA Craig T Dreyfus D Hsieh F The diagnosis and management of acute and chronic urticaria: 2014 update. J Allergy Clin Immunol (2014) 133:1270–7. 10.1016/j.jaci.2014.02.036 24766875

[41] Chang TW Chen C Lin CJ Metz M Church MK Maurer M The potential pharmacologic mechanisms of omalizumab in patients with chronic spontaneous urticaria. J Allergy Clin Immunol (2014) 135(2):337–342.e2. 10.1016/j.jaci.2014.04.036 24948369

[42] Lin H Boesel KM Griffith DT Prussin C Foster B Romero FA Omalizumab rapidly decreases nasal allergic response and FcepsilonRI on basophils. J Allergy Clin Immunol (2004) 113:297–302. 10.1016/j.jaci.2003.11.044 14767445

[43] Beck LA Marcotte GV MacGlashan D Togias A Saini S. Omalizumab-induced reductions in mast cell Fcepsilon RI expression and function. J Allergy Clin Immunol (2004) 114:527–30. 10.1016/j.jaci.2004.06.032 15356552

[44] Sanjuan MA Sagar D Kolbeck R. Role of IgE in autoimmunity. J Allergy Clin Immunol (2016) 137(6):1651–61. 10.1016/j.jaci.2016.04.007 27264000

[45] Lacombe Barrios J Begin P Paradis L Hatami A Paradis J Des Roches A Anti-IgE therapy and severe atopic dermatitis: a pediatric perspective. J Am Acad Dermatol (2013) 69:832–4. 10.1016/j.jaad.2013.05.035 24124824

[46] Heil PM Maurer D Klein B Hultsch T Stingl G Omalizumab therapy in atopic dermatitis: depletion of IgE does not improve the clinical course – a randomized, placebocontrolled and double blind pilot study. J Dtsch Dermatol Ges (2010) 8(12):990–8. 10.1111/j.1610-0387.2010.07497.x 20678148

[47] Iyengar SR Hoyte EG Loza A Bonaccorso S Chiang D Umetsu DT Immunologic effects of omalizumab in children with severe refractory atopic dermatitis: a randomized, placebo-controlled clinical trial. Int Arch Allergy Immunol (2013) 162(1):89–93. 10.1159/000350486 23816920 PMC4161454

[48] Wang HH Li YC Huang YC Efficacy of omalizumab in patients with atopic dermatitis: a systematic review and meta-analysis. J Allergy Clin Immunol (2016) 138(6):1719–22.e1. 10.1016/j.jaci.2016.05.038 27543070

[49] Hotze M Baurecht H Rodríguez E Chapman-Rothe N Ollert M Fölster-Holst R Increased efficacy of omalizumab in atopic dermatitis patients with wild-type filaggrin status and higher serum levels of phosphatidylcholines. Allergy (2014) 69:132–5. 10.1111/all.12234 24111531

[50] Casale TB Condemi J LaForce C Nayak A Rowe M Watrous M Effect of omalizumab on symptoms of seasonal allergic rhinitis: a randomized controlled trial. JAMA (2001) 286:2956–67. 10.1001/jama.286.23.2956 11743836

[51] Nayak A Casale T Miller SD Condemi J McAlary M Fowler-Taylor A Tolerability of retreatment with omalizumab, a recombinant humanized monoclonal anti-IgE antibody, during a second ragweed pollen season in patients with seasonal allergic rhinitis. Allergy Asthma Proc (2003) 24(5):323–9. 14619332

[52] Casale TB Bernstein IL Busse WW LaForce CF Tinkelman DG Stoltz RR Use of an anti-IgE humanized monoclonal antibody in ragweed-induced allergic rhinitis. J Allergy Clin Immunol (1997) 100:110–21. 10.1016/S0091-6749(97)70202-1 9257795

[53] Adelroth E Rak S Haahtela T Aasand G Rosenhall L Zetterstrom O Recombinant humanized mAb-E25, an anti-IgE mAb, in birch pollen-induced seasonal allergic rhinitis. J Allergy Clin Immunol (2000) 106:253–9. 10.1067/mai.2000.108310 10932067

[54] Okubo K Ogino S Nagakura T Ishikawa T. Omalizumab is effective and safe in the treatment of Japanese cedar pollen-induced seasonal allergic rhinitis. Allergol Int (2006) 55:379–86. 10.2332/allergolint.55.379 17130680

[55] Okubo K Nagakura T. Anti-IgE antibody therapy for Japanese cedar pollinosis: omalizumab update. Allergol Int (2008) 57:205–9. 10.2332/allergolint.R-08-164 18724074

[56] Nagakura T Ogino S Okubo K Sato N Takahashi M Ishikawa T. Omalizumab is more effective than suplatast tosilate in the treatment of Japanese cedar pollen-induced seasonal allergic rhinitis. Clin Exp Allergy (2008) 38:329–37. 10.1111/j.1365-2222.2007.02894.x 18070163

[57] Ogino S Nagakura T Okubo K Sato N Takahashi M Ishikawa T. Re-treatment with omalizumab at one year interval for Japanese cedar pollen-induced seasonal allergic rhinitis is effective and well tolerated. Int Arch Allergy Immunol (2009) 149:239–45. 10.1159/000199719 19218816

[58] Chervinsky P Casale T Townley R Tripathy I Hedgecock S Fowler-Taylor A Omalizumab, an anti-IgE antibody in the treatment of adults and adolescents with perennial allergic rhinitis. Ann Allergy Asthma Immunol (2003) 91:160–7. 10.1016/S1081-1206(10)62171-0 12952110

[59] Tsabouri S Tseretopoulou X Priftis K Ntzani EE. Omalizumab for the treatment of inadequately controlled allergic rhinitis: a systematic review and meta-analysis of randomized clinical trials. J Allergy Clin Immunol Pract (2014) 2:332–40.e1. 10.1016/j.jaip.2014.02.001 24811026

[60] Kuehr J Brauburger J Zielen S Schauer U Kamin W Von Berg A Efficacy of combination treatment with anti-IgE plus standard immunotherapy in polysensitized children and adolescents with seasonal allergic rhinitis. J Allergy Clin Immunol (2002) 109:274–80. 10.1067/mai.2002.121949 11842297

[61] Rolinck-Werninghaus C Hamelmann E Keil T Kulig M Koetz K Gerstner B The co-seasonal application of anti-IgE after pre-seasonal specific immunotherapy decreases ocular and nasal symptom scores and rescue medication use in grass pollen allergic children. Allergy (2004) 59:973–9. 10.1111/j.1398-9995.2004.00552.x 15291906

[62] Casale TB Busse WW Kline JN Ballas ZK Moss MH Townley RG Omalizumab pretreatment decreases acute reactions after rush immunotherapy for ragweed-induced seasonal allergic rhinitis. J Allergy Clin Immunol (2006) 117:134–40. 10.1016/j.jaci.2005.09.036 16387596

[63] Massanari M Nelson H Casale T Busse W Kianifard F Geba GP Effect of pretreatment with omalizumab on the tolerability of specific immunotherapy in allergic asthma. J Allergy Clin Immunol (2010) 125:383–9. 10.1016/j.jaci.2009.11.022 20159249

[64] Soriano Gomis V Gonzalez Delgado P Niveiro Hernandez E Failure of omalizumab treatment after recurrent systemic reactions to bee-venom immunotherapy. J Investig Allergol Clin Immunol (2008) 18(3):225–6. 18564638

[65] Galera C Soohun N Zankar N Caimmi S Gallen C Demoly P. Severe anaphylaxis to bee venom immunotherapy: efficacy of pretreatment and concurrent treatment with omalizumab. J Investig Allergol Clin Immunol (2009) 19(3):225–9. 19610266

[66] Nadeau KC Schneider LC Hoyte L Borras I Umetsu DT Rapid oral desensitization in combination with omalizumab therapy in patients with cow’s milk allergy. J Allergy Clin Immunol (2011) 127:1622–4. 10.1016/j.jaci.2011.04.009 21546071 PMC3396422

[67] Schneider LC Rachid R LeBovidge J Blood E Mittal M Umetsu DT A pilot study of omalizumab to facilitate rapid oral desensitization in high-risk peanut allergic patients. J Allergy Clin Immunol (2013) 132:1368–74. 10.1016/j.jaci.2013.09.046 24176117 PMC4405160

[68] Lafuente I Mazon A Nieto M Uixera S Pina R Nieto A Possible recurrence of symptoms after discontinuation of omalizumab in anti-IgE-assisted desensitization to egg. Pediatr Allergy Immunol (2014) 25:717–9. 10.1111/pai.12259 24902874

[69] Bégin P Dominguez T Wilson SP Bacal L Mehrotra A Kausch B Phase 1 results of safety and tolerability in a rush oral immunotherapy protocol to multiple foods using omalizumab. Allergy Asthma Clin Immunol (2014) 10:7.52. 10.1186/1710-1492-10-7 24576338 PMC3936817

[70] Frischmeyer-Guerrerio PA Masilamani M Gu W Brittain E Wood R Kim J Mechanistic correlates of clinical responses to omalizumab in the setting of oral immunotherapy for milk allergy. J Allergy Clin Immunol (2017). 10.1016/j.jaci.2017.03.028 28414061 PMC5632581

[71] Leung DYM Sampson HA Yunginger JW Burks AW Schneider L Wortel CH Effect of anti-IgE therapy in patients with peanut allergy. N Engl J Med (2003) 348:986–93. 10.1056/NEJMoa022613 12637608

[72] Sampson HA Leung DY Burks AW Lack G Bahna SL Jones SM A phase II, randomized, double-blind, parallel-group, placebo-controlled oral food challenge trial of Xolair (omalizumab) in peanut allergy. J Allergy Clin Immunol (2011) 127:1309–10.e1. 10.1016/j.jaci.2011.01.051 21397314

[73] Rocha R Vitor AB Trindade E Lima R Tavares M Lopes J Omalizumab in the treatment of eosinophilic esophagitis and food allergy. Eur J Pediatr (2011) 170(11):1471–4. 10.1007/s00431-011-1540-4 21809010

[74] Clayton F Fang JC Gleich GJ Lucendo AJ Olalla JM Vinson LA Eosinophilic esophagitis in adults is associated with IgG4 and not mediated by IgE. Gastroenterology (2014) 147(3):602–9. 10.1053/j.gastro.2014.05.036 24907494

[75] Loizou D Enav B Komlodi-Pasztor E Hider P Kim-Chang J Noonan L A pilot study of omalizumab in eosinophilic esophagitis. PLoS One (2015) 10(3):e0113483. 10.1371/journal.pone.0113483 25789989 PMC4366078

[76] Foster B Foroughi S Yin Y Prussin C. Effect of anti-IgE therapy on food allergen specific T cell responses in eosinophil associated gastrointestinal disorders. Clin Mol Allergy (2011) 9(1):7. 10.1186/1476-7961-9-7 21527026 PMC3098204

[77] Foroughi S Foster B Kim N Bernardino LB Scott LM Hamilton RG Anti-IgE treatment of eosinophil-associated gastrointestinal disorders. J Allergy Clin Immunol (2007) 120(3):594–601. 10.1016/j.jaci.2007.06.015 17765756 PMC2768344

[78] Tanou K Zintzaras E Kaditis AG. Omalizumab therapy for allergic bronchopulmonary aspergillosis in children with cystic fibrosis: a synthesis of published evidence. Pediatr Pulmonol (2014) 49:503–7. 10.1002/ppul.22937 24167019

[79] Jat KR Walia DK Khairwa A Anti-IgE therapy for allergic bronchopulmonary aspergillosis in people with cystic fibrosis. Cochrane Database Syst Rev (2013) 9:CD010288 10.1002/14651858.CD010288.pub2 24043500

[80] Collins J Devos G Hudes G Rosenstreich D. Allergic bronchopulmonary aspergillosis treated successfully for one year with omalizumab. J Asthma Allergy (2012) 5:65–70. 10.2147/JAA.S34579 23204847 PMC3508546

[81] Gevaert P Calus L Van Zele T Blomme K De Ruyck N Bauters W Omalizumab is effective in allergic and nonallergic patients with nasal polyps and asthma. J Allergy Clin Immunol (2013) 131:110–6. 10.1016/j.jaci.2012.07.047 23021878

[82] Pinto JM Mehta N DeTineo M Wang J Baroody FM Naclerio RM. A randomized, double-blind, placebo-controlled trial of anti-IgE for chronic rhinosinusitis. Rhinology (2010) 48:318–24. 10.4193/Rhin09.144 21038023

[83] Penn R Mikula S. The role of anti-IgE immunoglobulin therapy in nasal polyposis: a pilot study. Am J Rhinol (2007) 4:428–32. 10.2500/ajr.2007.21.3060 17882911

[84] Sokol KC Ghazi A Kelly BC Grant JA. Omalizumab as a desensitizing agent and treatment in mastocytosis: a review of the literature and case report. J Allergy Clin Immunol Pract (2014) 2(3):266–70. 10.1016/j.jaip.2014.03.009 24811015

[85] Iglesias E Camacho Lovillo M Delgado Pecellín I Lirola Cruz MJ FalcónNeyra MD Salazar Quero JC Successful management of Churg-Strauss syndrome using omalizumab as adjuvant immunomodulatory therapy: first documented pediatric case. Pediatr Pulmonol (2014) 49:E78–81. 10.1002/ppul.22884 24136903

[86] Yu KK Crew AB Messingham KA Fairley JA Woodley DT. Omalizumab therapy for bullous pemphigoid. J Am Acad Dermatol (2014) 71:468–74. 10.1016/j.jaad.2014.04.053 24954907 PMC9215508

[87] Nonaka M Sakitani E Yoshihara T. Anti-IgE therapy to Kimura’s disease: a pilot study. Auris Nasus Larynx (2014) 41:384–8. 10.1016/j.anl.2013.12.006 24406057

[88] Bobolea I Barranco P Fiandor A Cabañas R Quirce S Omalizumab: a potential new therapeutic approach for aspirin-exacerbated respiratory disease. J Investig Allergol Clin Immunol (2010) 20(5):448–9. 20945617

[89] Lee J Successful prevention of recurrent anaphylactic events with antiimmunoglobulin E therapy. Asia Pac Allergy (2014) 4:126–8. 10.5415/apallergy.2014.4.2.126 24809019 PMC4005346

[90] Kupryś-Lipińska I Korczyńska P Tworek D Kuna P. Effectiveness of omalizumab in a patient with a life-threatening episode of bronchospasm and larynx angioedema after exposure to house dust. Postepy Dermatol Alergol (2014) 31:39–44. 10.5114/pdia.2014.40659 24683397 PMC3952055

[91] Ozturk AB Kocaturk E. Omalizumab in recurring larynx angioedema: a case report. Asia Pac Allergy (2014) 4:129–30. 10.5415/apallergy.2014.4.2.129 24809020 PMC4005344

[92] Kaya H Gümüş S Uçar E Aydoğan M Muşabak U Tozkoparan E Omalizumab as a steroid-sparing agent in chronic eosinophilic pneumonia. Chest (2012) 142:513–6. 10.1378/chest.11-1881 22871762

[93] de Klerk TA Sharma V Arkwright PD Biswas S. Severe vernal keratoconjunctivitis successfully treated with subcutaneous omalizumab. J AAPOS (2013) 17:305–6. 10.1016/j.jaapos.2012.12.153 23607979

